# Novel over‐the‐wire stent exchange technique with a loop cutter for endoscopic transpapillary gallbladder drainage in a complex anatomical case

**DOI:** 10.1111/den.14996

**Published:** 2025-01-20

**Authors:** Yasuhiro Kuraishi, Ichitaro Horiuchi, Akira Nakamura

**Affiliations:** ^1^ Department of Gastroenterology Shinshu University Hospital Nagano Japan

## Abstract

Watch a video of this article.

## BRIEF EXPLANATION

Endoscopic transpapillary gallbladder drainage (ETGBD) is effective for acute cholecystitis in patients contraindicated for emergency cholecystectomy, but poses several challenges, especially during guidewire advancement into the gallbladder.^1^, ^2^ Stent replacement for recurrent cholecystitis presents similar difficulties. Involving guidewire insertion through the existing stent followed by stent removal while maintaining guidewire access, the over‐the‐wire stent exchange technique is a viable strategy. We present the successful application of this technique in balloon enteroscopy‐assisted ETGBD.

**Figure 1:**
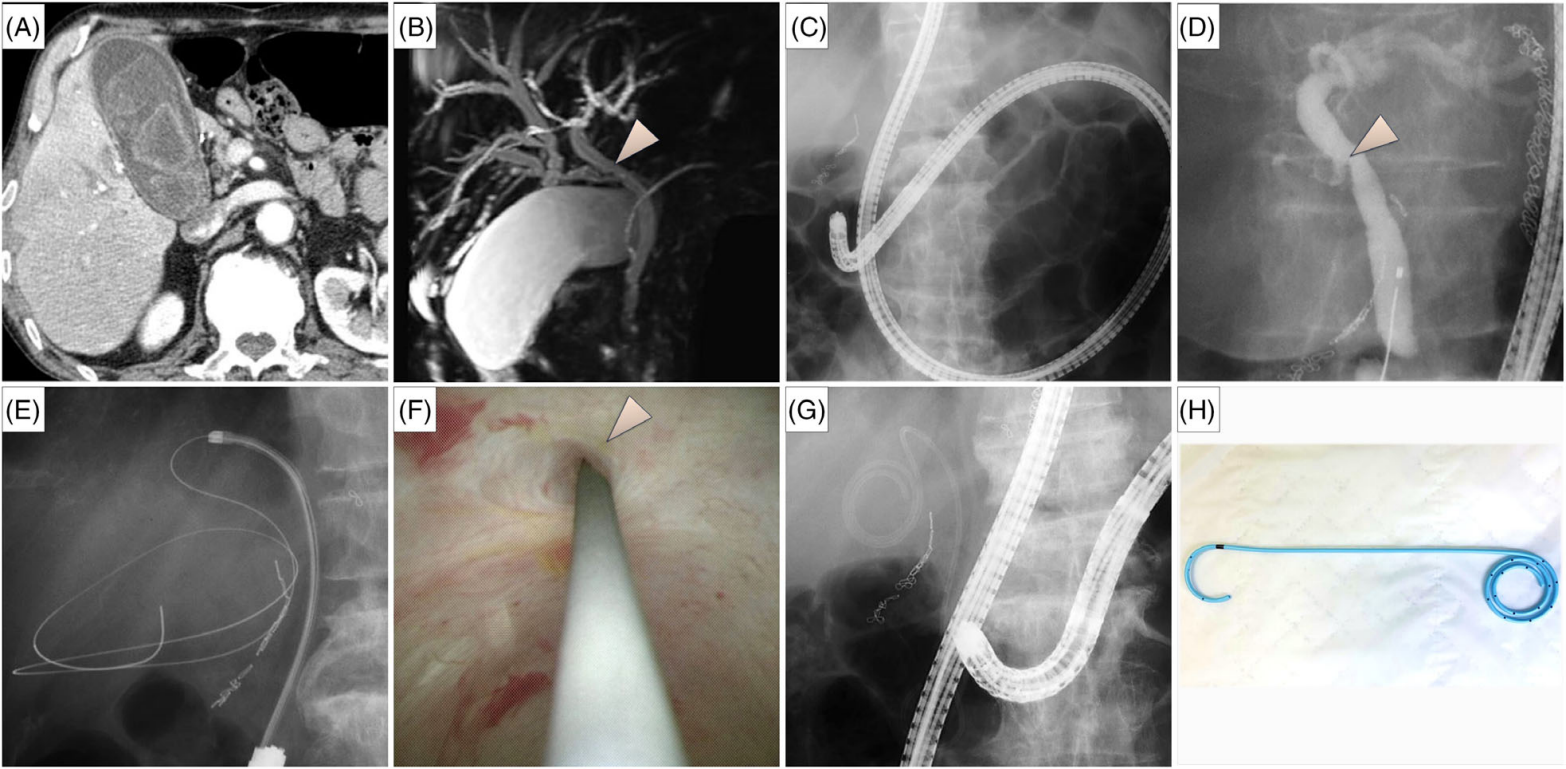
(A) Computed tomography showed findings consistent with acute cholecystitis, including gallbladder distention, wall thickening, and pericholecystic fluid. Multiple gallstones were present. (B) Magnetic resonance cholangiopancreatography revealed an anomalous junction in which the right hepatic duct joined the cystic duct. An arrowhead indicates the confluence of the cystic duct and common bile duct. (C) Endoscopic transpapillary gallbladder drainage was performed using a short‐type single‐balloon enteroscope (SIF‐H290; Olympus Medical Systems, Tokyo, Japan). (D) Cholangiography showed the caudal branching of the cholecystic duct indicative of challenging guidewire insertion into the gallbladder. (E,F) Guidewire access to the gallbladder was successful under cholangioscopic guidance. (G) A 5F plastic stent (IYO‐stent; Gadelius Medical, Tokyo, Japan) was placed into the gallbladder. (H) Specifically designed for transpapillary gallbladder drainage, this 5F stent features a proximal spiral structure with multiple side holes and a distal pigtail shape to prevent migration.

**Figure 2:**
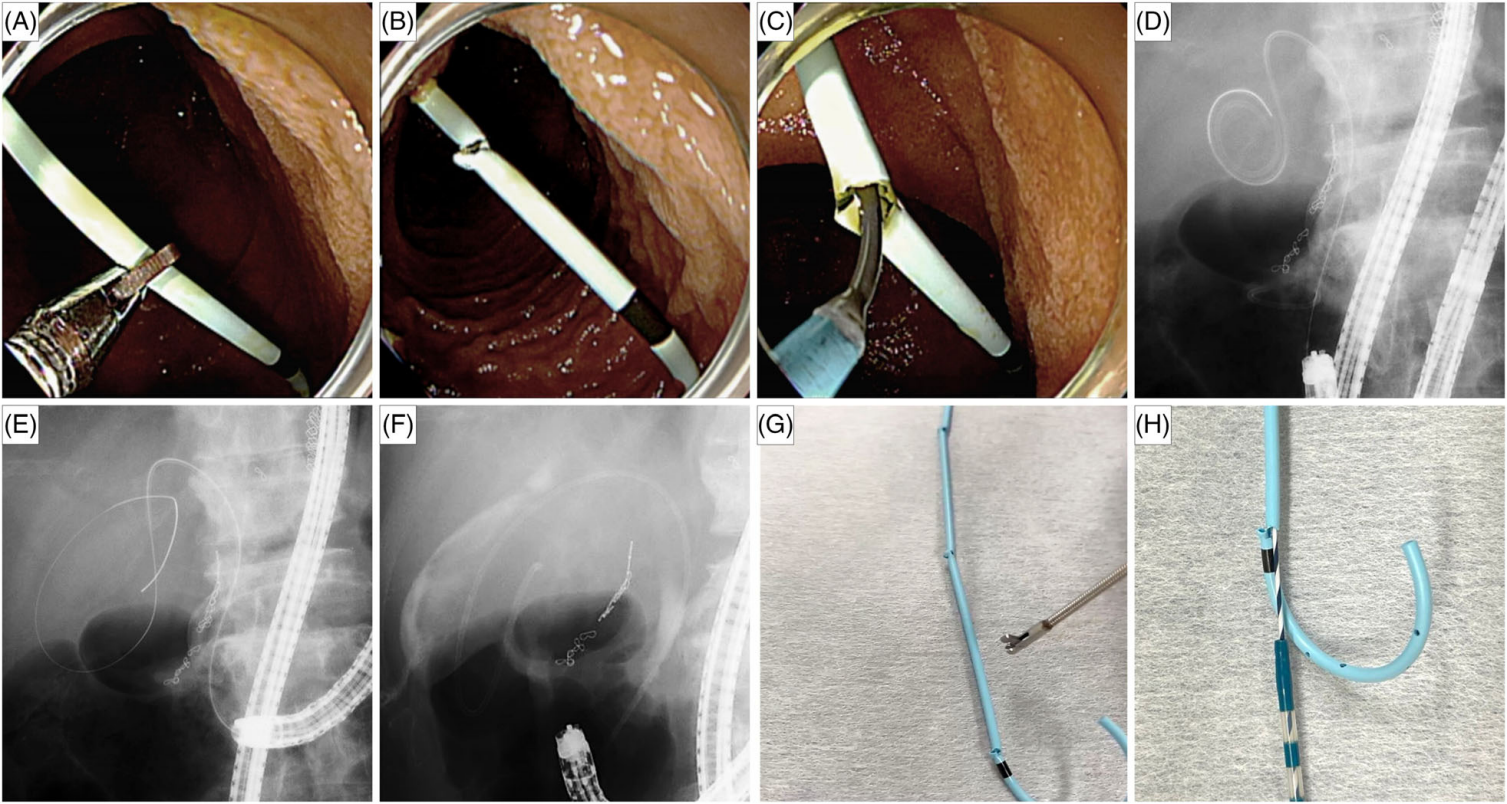
(A,B) The distal portion of the existing 5F stent body was cut using a loop cutter (FS‐5L‐1; Olympus Medical Systems, Tokyo, Japan) to create an access point opening to the gallbladder. (C) A guidewire was inserted through the opening created in the stent body. (D) The guidewire was advanced to the gallbladder through the stent lumen. (E) The stent was removed over the wire using forceps, leaving the guidewire within the gallbladder. (F) A new 5F plastic stent was placed into the gallbladder. (G,H) A bench test was conducted to assess the reproducibility and feasibility of creating access points in a 5F stent using a loop cutter. Multiple access holes were successfully created, demonstrating consistent performance without significant distortion or collapse of the stent body, and allowing guidewire insertion.

A 74‐year‐old man with prior total gastrectomy and Roux‐en‐Y reconstruction receiving immunosuppressive medications for severe rheumatoid arthritis presented with acute cholecystitis. Imaging revealed multiple gallstones and an anomalous junction of the right hepatic duct and cystic duct (Fig. 1). Given his poor surgical condition, we performed ETGBD using a short‐type single‐balloon enteroscope. Guidewire insertion under fluoroscopic guidance failed from caudal bifurcation of the cystic duct and limited scope maneuverability, necessitating cholangioscopic guidance for guidewire access. A 5F plastic stent (IYO‐stent; Gadelius Medical, Tokyo, Japan) with a proximal spiral structure and a distal pigtail^3^ was placed into the gallbladder. Three months later, recurrent cholecystitis required stent replacement (Fig. 2A–F; Video S1). We opted for the over‐the‐wire stent exchange technique knowing the guidewire access challenges encountered in the initial procedure. The pigtail configuration complicated guidewire insertion from the stent's distal end. A loop cutter (FS‐5L‐1; Olympus Medical Systems, Tokyo, Japan) was used to create an opening in the stent body to enable guidewire insertion. The stent was removed over the guidewire with forceps, and a new 5F plastic stent was placed.

A bench test confirmed the reproducibility of creating an access hole in the 5F plastic stent using a loop cutter without distortion or collapse (Fig. 2G,H). The over‐the‐wire stent exchange technique effectively facilitated ETGBD in patients with complex anatomy by enabling guidewire advancement via a loop cutter‐created access point in a 5F stent.

Authors declare no conflict of interest for this article.

## Supporting information


**Video S1** This case demonstrates the utility of the over‐the‐wire stent exchange technique in endoscopic transpapillary gallbladder drainage for patients with complex anatomy and difficult guidewire access. Using a loop cutter to create an access point in the existing 5F plastic stent enabled effective guidewire advancement.
